# Improving Olive Leaf Phenolic Extraction with Pulsed Electric Field Technology Pre-Treatment

**DOI:** 10.3390/foods14030368

**Published:** 2025-01-23

**Authors:** María del Carmen Razola-Díaz, Robert Sevenich, Oliver K. Schlüter, Vito Verardo, Ana María Gómez-Caravaca

**Affiliations:** 1 Department of Nutrition and Food Science, University of Granada, Campus of Cartuja s/n, 18071 Granada, Spain; carmenrazola@ugr.es (M.d.C.R.-D.); vitoverardo@ugr.es (V.V.); 2 Institute of Nutrition and Food Technology ‘José Mataix’, Biomedical Research Centre, University of Granada, Avda del Conocimiento s/n., 18100 Granada, Spain; 3 Leibniz-Institut für Agrartechnik und Bioökonomie e.V. (ATB), Max-Eyth-Alle 100, D-14469 Potsdam, Germany; 4 Department of Analytical Chemistry, Faculty of Sciences, University of Granada, Avda Fuentenueva s/n, 18071 Granada, Spain

**Keywords:** phenolic compounds extraction, olive leaves, non-thermal technology, hydroxytyrosol, oleuropein

## Abstract

The olive leaf is one of the main by-products from the olive oil industry. This by-product is a rich source of phenolic compounds that have been shown to possess beneficial health activities, which are due in part to their antioxidant activities. Therefore, the revaluation of this by-product would be of great importance for the food industry. For this reason, this study focuses on the pretreatment of olive leaves with a technology based on the use of pulsed electric fields (PEF) and their following extraction by ultrasounds in order to obtain an extract enriched in phenolic compounds. A Box-Behnken design of 15 experiments with three independent factors has been carried out: electric field strength (kV/cm), frequency (Hz) and total treatment time (s). The response variables were the sum of phenolic compounds, hydroxytyrosol and oleuropein measured by HPLC-MS-ESI-TOF and the antioxidant activity measured by DPPH. The validity of the experimental design was confirmed by ANOVA and the optimal conditions were established by using the response surface methodology in combination with a desirability function. The PEF optimal conditions were 0.6 kV/cm at 90 Hz for 11 s, which allowed for obtaining an olive leaf extract with 26.8, 21.7 and 15.6% higher contents of hydroxytyrosol, oleuropein and total phenolic compounds, respectively, compared to the non-treated sample with PEF. The antioxidant activity measured by DPPH was increased significantly by 32.3%. The data confirmed that the pre-treatment with PEF under these optimal conditions has proven to be effective in improving the extraction of phenolic compounds in olive leaves.

## 1. Introduction

The olive oil industry, a cornerstone of Mediterranean agriculture, produces substantial by-products, particularly olive leaves, which are generated during pruning and harvesting. They are not used and discarded or destined to animal feed [1]. These leaves are abundant in bioactive phenolic compounds, including hydroxytyrosol, oleuropein, and various flavonoids, which are renowned for their antioxidant, anti-inflammatory and antimicrobial properties, potentially offering cardiovascular, neuroprotective and anti-cancer health benefits [2,3]. The revalorization of these olive leaves aligns with sustainability goals in the food industry, providing a pathway for transforming agricultural waste into valuable bioactive ingredients for functional foods, nutraceuticals and cosmetics [4,5]. However, efficiently extracting these phenolic compounds presents challenges due to the robust cellular structure of the leaves, which limits the release of these bioactives during traditional extraction processes. Conventional extraction methods, such as solvent extraction and maceration, have been used to recover phenolics from olive leaves but are often limited by their need for high solvent volumes, prolonged extraction times and high temperatures, which can degrade sensitive phenolic compounds [6]. In addition to these limitations, solvent extraction processes can be costly and environmentally detrimental, thereby conflicting with industry trends toward greener, more sustainable practices [7]. As a result, non-thermal techniques, including Pulsed Electric Field (PEF) technology, have gained attention as promising alternatives that can preserve phenolic integrity, reduce solvent use and increase extraction yields [8]. Other non-conventional techniques have been previously applied in olive leaves to extract phenolic compounds such as deep eutectic solvents [9,10,11], supercritical CO_2_ [12], microwave assisted extraction [13] and enzyme-assisted extraction [14]. PEF technology, which involves applying short pulses of high-voltage electric fields to a material, induces electroporation—a process that permeabilizes cell membranes, enhancing the release of intracellular compounds such as phenolics [15,16]. This technique has demonstrated effectiveness in various food-processing applications, including microbial inactivation and extraction processes, particularly for compounds housed within robust cellular matrices [17,18]. The application of PEF in phenolic extraction has shown considerably promise, with studies reporting increased yields and improved bioavailability of phenolic compounds from a range of plant materials, including leaves [19,20,21,22,23,24] and other fruit by-products [25,26,27,28,29,30,31,32,33,34,35]. Moreover, research suggests that using PEF as a pre-treatment not only improves extraction yields but also shortens extraction times and reduces the need for extensive solvent use, making it an appealing technique from both an environmental and economic standpoint [36].

In this context, this study aims to establish optimal conditions for PEF pre-treatment for the ultrasound-assisted extraction of phenolic compounds, including hydroxytyrosol and oleuropein, with high antioxidant activity from olive leaves. For this, a Box-Behnken experimental design coupled with a response surface methodology and a quadratic desirability function were used for the optimization. The optimized PEF parameters included electric field strength, frequency and total operating time.

## 2. Materials and Methods

### 2.1. Reagents and Samples

Water was purified using a Milli-Q system (Millipore, Bedford, MA, USA). 2,2-diphenyl-1-picrylhydrazyl (DPPH), Trolox, hydroxytyrosol, oleuropein, chlorogenic acid, apigenin and rutin were acquired from Sigma-Aldrich (St. Louis, MO, USA). Other reagents were purchased from Merck KGaA (Darmstadt, Germany).

Olive leaves (*Olea europaea* L., cv. Arbequina) were provided by an olive oil producer of Granada (Spain) in January 2024.

### 2.2. Experimental Design

To optimize the PEF pre-treatment conditions for olive leaves, an experimental design was used to minimize the number of trials while ensuring the reliability of the experimental hypotheses. A Box-Behnken design consisting of 15 experiments, each replicated three times, was utilized. The independent variables included electric field strength (0.1, 0.6 and 1.1 kV/cm), frequency (50, 100 and 150) and total operating time (5, 10 and 15 s). The responses measured included hydroxytyrosol, oleuropein, total phenolic compounds determined by HPLC-MS and the antioxidant activity measured by DPPH. The dependent variables were fitted to a second-order polynomial model equation (Equation (1)), where Υ represents the response variables, X_i_ and X_j_ are the independent factors that affect the response and β_0_, β_i_, β_ii_ and β_ij_ are the regression coefficients of the model (interception, linear, quadratic and interaction terms).

Equation (1). Second-order polynomial equation.

(1) $$\Upsilon =\beta_{0}+\sum_{i=0}^{4}\beta_{i}X_{i}+\sum_{i=0}^{4}\beta_{ii}X_{ii}^{2}+\sum_{i=0}^{4}\sum_{j=0}^{4}\beta_{ii}X_{i}X_{j}$$

An ANOVA was conducted to assess the model fit, which included regression coefficients, *p*-values and an analysis of lack of fit. Additionally, response surface methodology (RSM) and a quadratic desirability function were used to determine the optimal conditions.

### 2.3. Pulse Electric Field Treatment

PEF pre-treatments were carried out in a pilot scale system (HVP 5 PEF system, ELEA, Quakenbrück, Germany) that can reach a maximum voltage of 20 kV. The electric field was applied to the samples in a chamber with a distance of 2 cm between the electrodes. For each experiment, 20 g of olive leaves were added to 60 g of tap water (~1 mS/cm). The pulse shape (square wave bipolar pulse) was monitored online using an oscilloscope during PEF treatment. At the end of the process, the olive leaves were filtrated and dried at room temperature.

For the experimental design, the pulse width was fixed to 15 µs for all the trials and the experiments were carried out following the conditions stated in Table 1, with specific energy input in the range of 0.39–7.71 kJ/kg.

### 2.4. Ultrasound-Assisted Extraction

Olive leaves, PEF pre-treated or non-PEF pre-treated (control) (0.5 g), were extracted with a 55/45 ethanol/water solution (*v*/*v*) (100 mL) by a sonotrode (UP400St ultrasonic processor, Hielscher, Germany) of 400 W and 24 kHz, equipped with an ultrasonic probe of 22 mm in diameter and an amplitude of 100% for 8 min, in duplicate. Those parameters were previously optimized [37]. After the extraction, the samples were centrifuged at 3500 rpm for 15 min, the supernatant was evaporated in a rotary evaporator and the extract was dissolved in 2 mL of MeOH/H_2_O 1/1 (*v*/*v*) and stored at −18 °C until the analysis.

### 2.5. Determination of Phenolic Compounds by HPLC-ESI-TOF-MS

The determination of the phenolic compounds of the olive leaves extracts was carried out as described by Talhaoui et al. (2014) [38]. Briefly, analyses were carried out in duplicate on an Agilent 1200 series Rapid Resolution Liquid Chromatograph (Agilent Technologies, Palo Alto, CA, USA) coupled to a micrOTOF (Bruker Daltonics, Bremen, Germany) orthogonal-accelerated time-of-flight (TOF) mass spectrometer equipped with an electrospray interface (ESI) (model G1607A, Agilent Technologies, Palo Alto, CA, USA), working in negative ionization mode. The system utilized a Poroshell 120 EC-C18 analytical column (4.6 × 100 mm, 2.7 µm) from Agilent Technologies. The mobile phases were water containing 1% acetic acid (phase A) and acetonitrile (phase B), with a solvent gradient programmed as follows: 0 min, 5% B; 4 min, 9% B; 7 min, 12% B; 8 min, 15% B; 9 min, 16% B; 14 min, 20% B; 15 min, 22% B; 18 min, 28% B; 19 min, 30% B; 20 min, 31% B; 21.5 min, 32% B; 23 min, 34% B; 24 min, 35% B; 25.5 min, 40% B; 27 min, 50% B; 30 min, 100% B; 35 min, 100% B; and 37 min, 5% B. The flow rate was maintained at 0.8 mL/min throughout the analysis. The column temperature was held at 25 °C, and the injection volume was set to 2.5 µL. Five calibration curves were constructed in order to quantify the phenolic compounds identified in the olive leaves. The standards were selected according to the availability of commercial standards and based on previous research in olive products and by-products [37,39,40,41] where phenolic compounds are quantified with their standards or with compounds with similar structures that belong to the same phenolic family. Hydroxytyrosol (y = 202.33x + 139.29; R^2^ = 0.9971), oleuropein (y = 737.42x + 518.16; R^2^ = 0.9935), chlorogenic acid (y = 54.743x − 188.81; R^2^ = 0.9979) apigenin (y = 658.96x + 1694.6; R^2^ = 0.9911) and rutin (y = 411.18x + 300.08; R^2^ = 0.9922) were used as standards for quantification. This approach, although not providing absolute quantification for each individual compound, is consistent with methods reported in similar studies and ensures consistency in the relative comparisons between PEF-treated and control samples. The data were elaborated using MassLynx 4.1 software (Waters Corporation, Milford, MA, USA). Each extract was injected three times.

### 2.6. Determination of Antioxidant Activity

The antioxidant activity of the extracts was performed by DPPH radical scavenging activity with a method proposed by several authors [42,43]. Then, 100 µL of each extract was added to 2.9 mL of DPPH, and after rapid stirring, the bleaching power of the extract was observed in a time interval from 0 to 30 min at 517 nm. The results were compared with a standard curve of Trolox in methanol/water (4:1, *v*/*v*) (1, 10, 20, 50, 80, 100, 150, 200 µg/mL). Analyses were performed in triplicate. The results are expressed as mg Trolox equivalents (TE)/g d.w.

### 2.7. Statistical Analysis

Percentages were calculated as “100 − (control value × 100/treated value)” to represent the increase in phenolic content relative to the untreated control. The mathematical computations and simulations and ANOVA were performed using the Statistica 7.0 software package (StatSoft, Tulsa, OK, USA).

## 3. Results and Discussion

### 3.1. Fitting the Model

Exposure to an external electric field induces membrane electroporation in cells, increasing permeability and enhancing compound release during the extraction [44]. Therefore, finding optimal PEF conditions is essential for effective food-processing applications. PEF pre-treatment was optimized for extracting phenolic compounds, including hydroxytyrosol and oleuropein, from the olive leaf using a Box-Behnken design (Table 1).

**Table 1:** Box-Behnken experimental design for optimizing the PEF pre-treatment in olive leaves. Different letters (a–e) indicate significant differences among samples (*p* < 0.05).

	Independent Factors			Response Variables			
N.	Electric Field (kV/cm) X1	Frequency (Hz) X2	Total Operating Time (s) X3	Hydroxytyrosol (mg/g d.w.)	Sum of Oleuropein (mg/g d.w.)	Sum of Phenolic Compounds (mg/g d.w.)	DPPH (mg TE/g d.w.)
1	0.1	50	10	0.52 ± 0.03 b	8.00 ± 0.07 b	55.17 ± 2.13 b	27.07 ± 0.44 c,d
2	1.1	50	10	0.48 ± 0.01 b,c	7.90 ± 0.39 b	52.16 ± 4.71 b,c	26.07 ± 0.38 d
3	0.1	150	10	0.47 ± 0.03 b–d	6.23 ± 0.33 c,d	45.50 ± 2.32 c–e	26.54 ± 0.84 c,d
4	1.1	150	10	0.43 ± 0.02 c,d	4.82 ± 0.29 e	42.22 ± 3.49 d,e	29.72 ± 0.99 b–d
5	0.1	100	5	0.46 ± 0.04 b–d	5.23 ± 0.35 d,e	42.12 ± 1.90 d,e	30.51 ± 0.33 b–d
6	1.1	100	5	0.51 ± 0.01 b	7.70 ± 0.36 b	50.20 ± 2.21 b–d	30.78 ± 0.16 b–d
7	0.1	100	15	0.40 ± 0.02 d,e	7.19 ± 0.70 b,c	47.07 ± 2.19 b–d	36.79 ± 0.23 a,b
8	1.1	100	15	0.35 ± 0.02 e	5.20 ± 0.21 d,e	37.77 ± 2.96 e	30.56 ± 0.65 b–d
9	0.6	50	5	0.41 ± 0.01 d,e	6.07 ± 0.15 c,d	41.96 ± 1.43 d,e	31.23 ± 0.68 b–d
10	0.6	150	5	0.42 ± 0.00 c,d	6.28 ± 0.34 c,d	43.98 ± 3.26 c–e	33.32 ± 0.93 b,c
11	0.6	50	15	0.42 ± 0.04 c–e	7.70 ± 0.44 b	48.58 ± 1.20 b–d	30.92 ± 0.81 b–d
12	0.6	150	15	0.47 ± 0.03 b–d	7.08 ± 0.59 b,c	48.41 ± 4.01 b–d	33.64 ± 0.51 b,c
13	0.6	100	10	0.65 ± 0.00 a	9.59 ± 0.13 a	65.43 ± 3.13 a	41.61 ± 0.35 a
14	0.6	100	10	0.66 ± 0.00 a	9.78 ± 0.19 a	65.87 ± 1.85 a	41.96 ± 0.61 a
15	0.6	100	10	0.65 ± 0.04 a	9.51 ± 0.64 a	66.14 ± 5.63 a	41.58 ± 0.59 a

The evaluated responses were the content of hydroxytyrosol, the content of total oleuropein, the sum of phenolic compounds and the antioxidant activity measured by DPPH. Different results were obtained depending on the level of the electric field, frequency and total operating time applied to the olive leaves. Higher electric field strengths (1.1 kV/cm) generally correlated with lower hydroxytyrosol and total phenolic compound yields. Runs with high frequencies (150 Hz) and pulse numbers (1500 pulses) seemed to reduce the yield of hydroxytyrosol and oleuropein, suggesting that higher pulse numbers and frequencies might overprocess the sample, diminishing certain compounds’ stability or bioavailability. Moderate frequency settings (100 Hz) coupled with intermediate pulse numbers (1000 pulses) tended to yield higher phenolic contents and antioxidant activity. For example, entries 13–15 (0.6 kV/cm, 100 Hz, 10 s, 1000 pulses) displayed the highest hydroxytyrosol, oleuropein, and phenolic compound concentrations, alongside a stable antioxidant activity of around 41.61 mg TE/g d.w. Shorter operating times (5 s) generally yielded relatively stable antioxidant activity, without drastically reducing the phenolic content. For instance, entry 6 (1.1 kV/cm, 100 Hz, 5 s) showed a desirable balance with 50.20 mg/g d.w. phenolics and antioxidant activity at 30.78 mg TE/g d.w. Longer times (10 to 15 s), when combined with intermediate electric field strengths and frequency levels, maximized the phenolic yield and DPPH activity. The results highlight that moderate electric field and frequency levels with controlled pulse counts offered a balanced approach for maximizing the evaluated bioactive compound extraction and antioxidant potential.

Thus, data obtained experimentally were fitted to a second-order polynomial equation, and the regression coefficients are presented in Table 2. Using a significance level of *p* < 0.05, the non-significant terms were discarded, readjusting the model.

As can be seen in Table 2, the linear term **β_2_** (frequency), all the quadratic terms (**β_11_**, **β_22_** and **β_33_**) and the crossed term **β_13_** showed significance for all the response variables evaluated. In addition, the linear term **β_1_** (electric field) showed a significant effect for hydroxytyrosol and the sum of phenolic compounds, and **β_3_** (total operating time) did for hydroxytyrosol and DPPH. In case of the crossed terms, the interaction between frequency and electric field (**β_12_**) was also significant for the sum of oleuropein and DPPH, and the interaction between electric field and total treatment time (**β_23_**) was for hydroxytyrosol. Regarding the effects, although most of the linear and crossed terms showed a negative effect, all the quadratic terms had a positive effect. This highlights the importance of not only considering the linear and direct effect between samples but also the quadratic interactions [45]. A strong correlation between the dependent variables and factors was also noted, with R^2^ values > 0.9 in all cases. Model validity was confirmed by ANOVA, indicating a non-significant lack of fit (*p* > 0.05).

### 3.2. Optimization of PEF Pre-Treatment Conditions and Comparison Between PEF Pre-Treated and Control Olive Leaf

The response surface graphs generated were examined to identify optimal conditions for olive leaves, aiming to balance the evaluated factors and produce extracts with the highest possible bioactive content.

**Figure 1:**
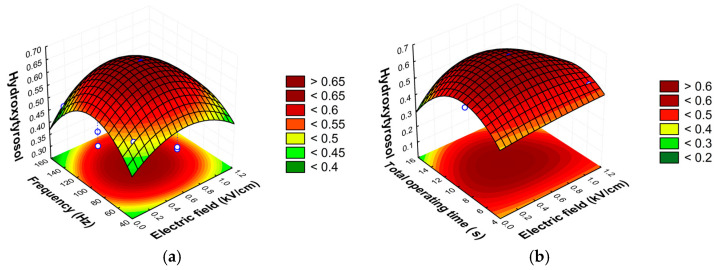

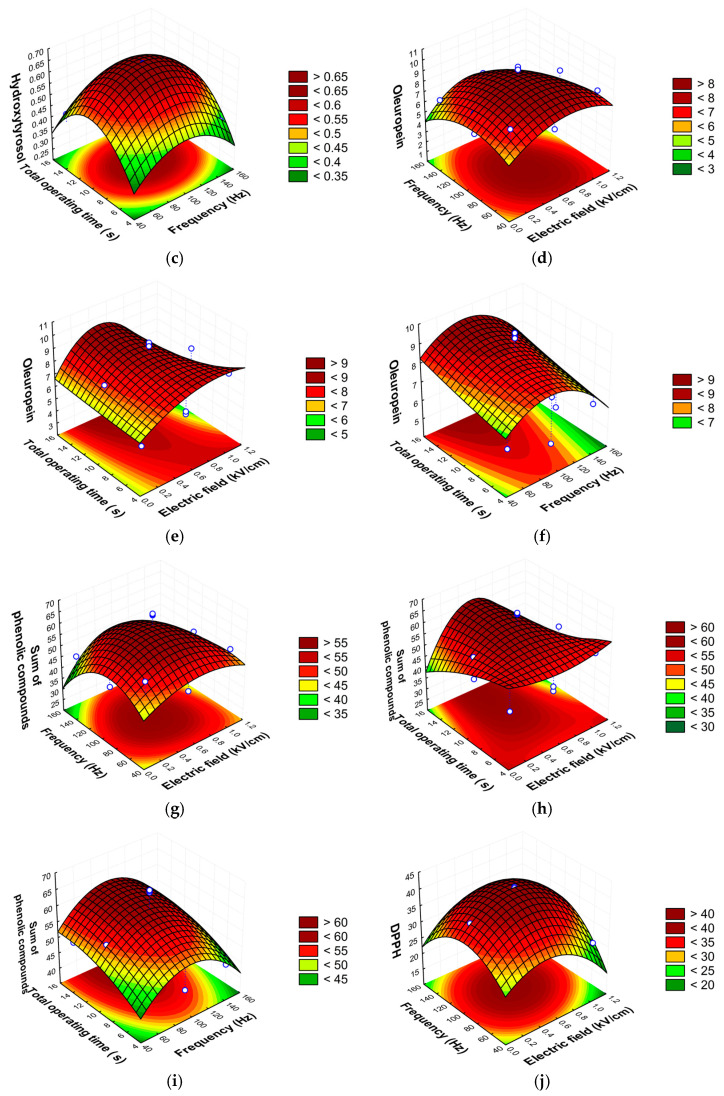

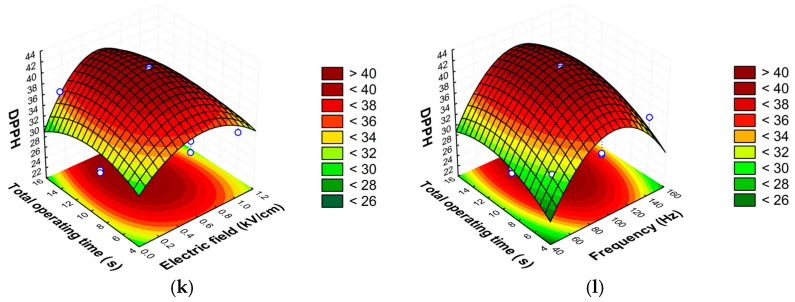
Response surface graphs showing the combined effects of process variables for hydroxytyrosol (**a**–**c**), oleuropein (**d**–**f**), the sum of phenolic compounds (**g**–**i**) expressed as mg/g d.w., and the antioxidant activity by DPPH (**j**–**l**) expressed as mg TE/g d.w.

The findings from the response surface graphs in Figure 1 provide valuable insights into the impact of PEF process factors on the extraction of hydroxytyrosol, oleuropein, total phenolic compounds, and antioxidant activity (DPPH). For hydroxytyrosol, the sum of phenolic compounds, and the antioxidant activity measured by DPPH, the results suggest that a balanced application of electric field strength, frequency and total operating time is essential for maximizing the yield. Moderate process intensities seem to promote extraction by increasing membrane permeability through electroporation, though overly intense conditions may degrade the compounds, reducing the antioxidant activity. In contrast, oleuropein responds slightly differently to these factors, with a particular sensitivity to electric field strength and frequency. Optimal oleuropein yields occur under moderate field strengths and frequencies but higher total operating times, implying that careful control is necessary to prevent the degradation that could occur under harsher conditions. In general, this trend suggests that moderate conditions promote phenolic extraction without significant degradation, allowing for the effective recovery of these compounds.

The model was processed and fitted to a quadratic function to predict optimal parameters for maximizing hydroxytyrosol, oleuropein, the sum of phenolic compounds and DPPH responses. To achieve this, response results were normalized on a dimensionless scale from 0 to 1, enabling the combination of factors with varying units or scales. This approach was integrated with response surface methodology for enhanced accuracy in optimization. The optimal settings identified for the olive leaves included an electric field of 0.6 kV/cm, a frequency of 90 Hz and a total operating time of 11, yielding a desirability score of 0.9641. The verification of the mathematical model showed that the predicted values closely matched the actual experimental results under optimal conditions, with coefficients of variation remaining below 10% for all the response variables. Additional parameters derived from these optimized conditions are provided in Table 3. Apart from these parameters, there are others that are also relevant when reporting a PEF treatment [46]; they were a specific energy input of 5.3 kJ/kg and 990 pulses of 15 µs.

The phenolic profile of the PEF pre-treated and control olive leaf extracts was extensively characterized, and the results are shown in Table 4. The concentrations reported in Table 4 should be interpreted as estimates, primarily intended to demonstrate the relative efficiency of PEF treatment.

Several studies have reported the extraction of phenolic compounds from olive leaves using different technologies such as conventional solvent extraction, ultrasound-assisted extraction, microwave-assisted extraction, supercritical fluid extraction, infrared-assisted extraction or pressurized liquid extraction [47]. However, there are few research studies regarding the use of PEF in olive leaves.

Pappas and co-workers [48] reported that using PEF (pulse duration: 10 µs, frequency 1000 Hz, electric field strength: 1 kV/cm and induction time: 30 min) as a pre-treatment for static solid–liquid extraction in 25% ethanol increased the total yield in polyphenols by up to 31.85%. They reported using a range of 0.155 to 1.55 kJ/kg of specific energy input and total treatment times in the range of 18 to 180 s [48]. Although using similar electric field strength and pulse width, in our case, a higher specific energy input (5.3 kJ/kg) and shorter total treatment time (11 s) were needed. They did not carry out a mathematical optimization, and the phenolic content was measured by the Folin–Ciocalteu spectrophotometric method and using maceration as an extraction technique and no other innovative technique as ultrasounds.

Tsevdou and co-workers [49] applied an electric field of 4.5 kV/cm, 5000 pulses (pulse width 15 µs) and a frequency of 5 Hz, resulting in a specific energy input of 175 kJ/kg, followed by one extraction by maceration with stirring and methanol 60% in olive pomace; they reported contents of 2 and 15.7 mg/g of hydroxytyrosol and total phenols, respectively [49]. Despite being a different olive by-product, it is noticeable that a significantly higher energy input was needed than for the olive leaves. They used maceration as an extraction technique with methanol, requiring more time.

PEF has also been applied in other leaves for extracting phenolic compounds. As described by Raso et al., the electric field strength and the specific energy input are parameters that can be used for comparing different treatments [46]. Some authors reported 2 kV/cm and 45 kJ/kg in custard apple leaves [19], 1 kV cm and 1.55 kJ/kg in salvia leaves [20], 5kV/cm and 6.18 kJ/kg in borage leaves [23] or 3 kV/cm and 4.1 kJ/kg in spearmint [24]. Other authors only reported the electric field strength being 0.75 kV/cm in moringa [22] and almon red leaves [50] or 0.6 kV/cm in laurel [51]. The optimized PEF conditions in olive leaves have been an electric field strength of 0.6 kV/cm and a specific energy input of 5.3 kJ/kg. Although some of the mentioned authors did not perform the optimization of the PEF technology, it can be affirmed that our optimized conditions are in the range of the conditions used in other leaves. The differences should be attributed to the other PEF parameters, the extraction technique used and the intrinsic structural differences.

There are a few previous research studies combining pulse electric fields with ultrasound extraction in leaves. Only Tzima and co-workers [52] applied this combination to rosemary and thyme. For the PEF pre-treatment, they used 167 bipolar pulses of a 30 μs pulse duration at 1.1 kV cm with specific energy inputs of 0.36 and 0.46 kJ/kg for rosemary and thyme, respectively, and the ultrasound extraction was carried out with the same ultrasound equipment used in the present study, but with a 22 mm titanium sonotrode horn tip model H22 in a sample/solvent ratio of 5/100 with 55.19% aqueous EtOH, 50% amplitude and 50% pulse for 12.48 min. They reported significant increments in both cases compared to not using the PEF as a pre-treatment [52]. The combination of PEF and ultrasounds was reported in orange peel [25], grape stems [29] and avocado peel and seed [26], with significant increments of 27.5, 35, 31.5 and 9.5%, respectively, compared to extracting with ultrasound extraction without PEF. In our case, with the optimal PEF conditions combined with ultrasound extraction, significant (*p* < 0.05) increments of 26.8, 21.7 and 15.6% were achieved in the hydroxytyrosol, oleuropein and total phenolic compounds contents in olive leaves. The antioxidant activity measured by DPPH increased significantly (32.3%).

The content of all these quantified compounds reported previously by other authors in olive leaves is in a wide range, attributed to the differences in olive leaf variety and origin [53] and the extraction technique [54]. Because of this, the obtained extract using the PEF optimal conditions was compared to a control extracted by the same procedure but not using PEF as a pre-treatment.

As can be seen in Table 4, an increase in all identified phenolic compounds was achieved in the PEF pre-treated olive leaf extract in comparison with the control extracted only with ultrasound without PEF. This could be due to several factors associated with how PEF impacts cell structures and facilitates the release of bioactive compounds. PEF treatment temporarily electroporates the cell membranes of plant material, a process known as electroporation. This allows for a more efficient release of intracellular compounds, including phenolics, during subsequent extraction [16]. PEF creates pores in cell membranes, which makes it easier for solvents to penetrate cells and for phenolic compounds to diffuse out. Since phenolic compounds are often stored in vacuoles within plant cells, breaking down these barriers allows the ultrasound extraction to be more effective, resulting in higher phenolic yields. Otherwise, PEF pre-treatment reduces the resistance to mass transfer, which means the phenolic compounds can move more readily from the cell interior to the extraction medium. This effect reduces the time and energy needed to release phenolics and increases the overall extraction efficiency [16]. In addition, when used in combination, PEF and ultrasound can have a synergistic effect. PEF first disrupts the cell structures, and then ultrasound, with its cavitation effect, provides an additional mechanical force that further breaks down cell walls, aiding in the release of phenolics [55]. Compared to other extraction methods that rely on heat, PEF can potentially reduce the oxidation and thermal degradation of phenolic compounds, preserving the stability and quantity of phenolics in the final extract [16].

The phenolic compounds profile in the olive leaf extracts was the same in the PEF pre-treated and non-pre-treated extracts. The quantified compounds can be classified in sericoidoids, flavonoids, hydroxytyrosol and derivatives and phenolic acids.

**Secoiridoids**, representing 70% of the phenolic compounds in the analyzed olive leaves, were the major phenolic group quantified. This group included oleoside, secologanoside, ligstroside, lucidumoside C, oleuropein and their derivatives. The total secoiridoid concentration was 41.6% higher in the PEF pre-treated extract (44.46 ± 4.04 mg/g) compared to the control (25.97 ± 1.99 mg/g). Among these, **oleuropein** is known for its antioxidant, anti-inflammatory and cardiovascular benefits [56]. This compound was increased by 21.7% in the PEF pre-treated extract (10.04 ± 0.96 mg/g) compared to the control (7.86 ± 0.72 mg/g). Notably, the oleuropein aglycon form, often linked to enhanced bioavailability and effectiveness, almost doubled the control (44.1% higher). Other oleuropein derivatives such as 2”-methoxyoleuropein, hydroxyoleuropein and oleuropein diglucoside were also found in significantly higher concentrations in the PEF-treated olive leaf compared to the control. This significant boost in sericoidoids suggests the optimized extraction enhances the therapeutic potential of the olive leaf extract by maximizing key compounds [56].

**Flavonoids** represented 16% of the phenolic compounds quantified in the olive leaves extracts. Their levels were also significantly higher (10.57 ± 0.92 mg/g), with a 36.5% increase over the control (6.71 ± 0.63 mg/g). This group included quercetin, diosmin, rutin, taxifolin, chrysoeriol glucoside, apigenin derivatives (apigenin rutinoside and apigenin glucoside), luteolin and its derivatives (luteolin rutinoside, luteolin glucoside and luteolin diglucoside) and taxifolin. Among them, the major ones were luteolin glucoside and chrysoeriol glucoside, with significant increments of 4.9 and 58.1%, respectively, compared to the control.

Other minor groups were quantified in the olive leaves. The caffeic acid derivative forms represented 7% of all the phenolic compounds quantified in the olive leaves, with a total increase of 49% in the PEF pre-treated ones (4.02 ± 0.25 mg/g) compared to the control (2.05 ± 0.06 mg/g).

**Hydroxytyrosol**, alongside its derivative **verbascoside**, were also quantified in the olive leaves, representing 7% of all the phenolic compounds. Hydroxytyrosol is known for its wide range of biological activities, including anti-inflammatory, anti-tumor, antiviral, antibacterial and antifungal effects. It also plays a role in enhancing endothelial function, reducing oxidative stress and offering protection to both the nervous and cardiovascular systems. Thanks to these health-promoting attributes, hydroxytyrosol is currently one of the most extensively studied natural phenolic compounds [57]. It is the first approved phenolic ingredient in the EU and may be incorporated into various food products [58]. Because of this, hydroxytyrosol was one of the compounds for which PEF optimization was focused on and was increased by 26.8% compared with the control. Otherwise, verbascoside is a phenolic glucoside derived from phenylpropanoids, specifically from the compounds caffeic acid and hydroxytyrosol. Its structure combines a phenylpropanoid backbone with sugars (glucose and rhamnose), creating a complex molecule that gives it antioxidant and anti-inflammatory properties [59]. It achieved an increment of 53% compared to the olive leaf extracted without PEF as a pre-treatment.

## 4. Conclusions

In conclusion, this study confirmed that PEF pre-treatment followed by ultrasound extraction significantly enhances phenolic compounds from olive leaves, yielding higher concentrations across major phenolic groups than not using PEF. Under the optimal conditions, this study achieved a 26.8% increase in hydroxytyrosol, a 21.7% increase in oleuropein and a 15.6% increase in the total phenolic content compared to untreated samples. Additionally, antioxidant activity, as measured by DPPH, increased by 32.3%, highlighting the impact of PEF on enhancing the therapeutic and functional value of olive leaf extracts. Secoiridoids, particularly oleuropein and its derivatives, as well as flavonoids, phenolic acids, hydroxytyrosol and verbascoside, showed high gains in concentration, further indicating the effectiveness of PEF in releasing these bioactives. Given the well-documented antioxidant, anti-inflammatory and cardioprotective effects of these compounds, the PEF-enhanced extraction method not only maximizes the therapeutic potential of olive leaf extracts but also offers a promising approach for future applications in food and nutraceutical industries. However, the responses of different olive leaf varieties to PEF treatment may vary, impacting the generalizability of the findings. Future research should investigate the cost–benefit analysis of PEF technology in commercial production and evaluate its efficacy across diverse olive cultivars.

## Figures and Tables

**Table 2:** Regression coefficients effect of PEF pre-treatment optimization model in olive leaf.

Regression Coefficients	Hydroxytyrosol (mg/g d.w.)	Sum of Oleuropein (mg/g d.w.)	Sum of Phenolic Compounds (mg/g d.w.)	DPPH (mg TE/g d.w.)
**β_0_**	0.44 *	6.62 *	46.26 *	30.60 *
**Linear**				
**β_1_**	−0.013 *	−0.21	−1.15 *	−0.13
**β_2_**	−0.011 *	−0.84 *	−3.12 *	0.92 *
**β_3_**	−0.033 *	0.11	−0.32	1.01 *
**Quadratic**				
**β_11_**	0.044 *	0.84 *	4.62 *	3.62 *
**β_22_**	0.046 *	0.61 *	3.90 *	3.56 *
**β_33_**	0.067 *	0.81*	6.14 *	1.16 *
**Crossed**				
**β_12_**	0.00052	−0.33 *	−0.066	1.05 *
**β_13_**	−0.028 *	−1.12 *	−4.35 *	−1.62 *
**β_23_**	0.0086 *	−0.21	−0.55	0.16
**R^2^**	0.9993	0.9397	0.9948	0.9953
***p* lack of fit**	0.7636	0.0813	0.1661	0.1906

* significant at *p* < 0.05.

**Table 3:** Optimized and other parameters of PEF pre-treatment with optimal results in olive leaf.

**Optimized Parameters**				
**Field strength (kV/cm), X_1_**	0.6			
**Frequency (Hz), X_2_**	90			
**Total operating time (s), X_3_**	11			
**Other Parameters**				
**Specific energy (kJ/kg)**	5.3			
**Pulse width (µs)**	15			
**Total treatment time (ms)**	14.8			
**Number of pulses**	990			
**Voltage (V)**	1200			
	**Hydroxytyrosol**	**Oleuropein**	**Sum of Phenolic** **Compounds**	**DPPH**
**Desirability value**	0.9641			
**Predicted value** **(mg/g d.w.)**	0.64 ± 0.91	9.67 ± 0.48	64.49 ± 1.24	41.09 ± 0.74
**Obtained value** **(mg/g d.w.)**	0.67 ± 0.05 a	10.04 ± 0.96 a	63.94 ± 5.57 a	42.32 ± 0.46 a
**Control (mg/g d.w.)**	0.49 ± 0.04 b	7.86 ± 0.72 b	53.95 ± 3.82 b	28.26 ± 1.11 b

Different letters (a,b) in the same column indicate significant (*p* < 0.05) differences.

**Table 4:** HPLC-ESI-TOF-MS quantified phenolic compounds in optimum and control olive leaf extracts (mg/g d.w.) expressed as average ± standard deviation.

Compound	Optimum	Control
2″-Methoxyoleuropein isomer a	2.06 ± 0.19 a	1.50 ± 0.10 b
2″-Methoxyoleuropein isomer b	2.18 ± 0.20 a	1.30 ± 0.00 b
2″-Methoxyoleuropein isomer c	0.77 ± 0.07	<LOQ
Apigenin glucoside	0.55 ± 0.05 a	0.53 ± 0.05 a
Apigenin rutinoside	0.26 ± 0.03 a	0.20 ± 0.02 b
Caffeic acid derivate isomer a	0.56 ± 0.04 a	0.40 ± 0.03 b
Caffeic acid derivate isomer b	0.65 ± 0.06	<LOQ
Caffeic alcohol derivative	2.81 ± 0.15 a	1.65 ± 0.02 b
Chrysoeriol glucoside isomer a	0.94 ± 0.08 a	0.30 ± 0.03 b
Chrysoeriol glucoside isomer b	1.52 ± 0.15 a	0.73 ± 0.07 b
Demethyloleuropein	0.43 ± 0.00 a	0.21 ± 0.02 b
Diosmin	0.36 ± 0.03 a	0.10 ± 0.01 b
Hydroxyoleuropein isomer a	0.99 ± 0.10 a	0.71 ± 0.07 b
Hydroxyoleuropein isomer b	0.90 ± 0.09 a	0.61 ± 0.06 b
Hydroxyoleuropein isomer c	0.67 ± 0.06	<LOQ
Hydroxyoleuropein isomer d	0.46 ± 0.05	<LOQ
Hydroxytyrosol	0.67 ± 0.05 a	0.49 ± 0.04 b
Ligstroside isomer a	0.26 ± 0.03	<LOQ
Ligstroside isomer b	1.03 ± 0.10 a	0.83 ± 0.07 b
Lucidumoside C isomer a	1.74 ± 0.12 a	1.21 ± 0.11 b
Lucidumoside C isomer b	1.76 ± 0.17 a	1.31 ± 0.12 b
Lucidumoside C isomer c	0.50 ± 0.05	<LOQ
Lucidumoside C isomer d	2.50 ± 0.23	<LOQ
Luteolin	0.68 ± 0.00	<LOQ
Luteolin diglucoside	0.75 ± 0.07 a	0.31 ± 0.03 b
Luteolin glucoside isomer a	0.98 ± 0.09 a	1.12 ± 0.11 a
Luteolin glucoside isomer b	0.08 ± 0.01 a	0.11 ± 0.01 a
Luteolin glucoside isomer c	1.01 ± 0.10 a	1.04 ± 0.10 a
Luteolin glucoside isomer d	0.54 ± 0.05 a	0.24 ± 0.02 b
Luteolin rutinoside	0.11 ± 0.01 a	0.05 ± 0.00 b
Oleoside	1.21 ± 0.11 a	0.81 ± 0.02 b
Oleoside methyl ester isomer a	0.67 ± 0.07 a	0.42 ± 0.01 b
Oleoside methyl ester isomer b	0.78 ± 0.07 a	0.36 ± 0.04 b
Oleuropein aglycon	4.72 ± 0.44 a	2.64 ± 0.25 b
Oleuropein diglucoside isomer a	0.61 ± 0.06 a	0.26 ± 0.03 b
Oleuropein diglucoside isomer b	0.52 ± 0.05 a	0.25 ± 0.02 b
Oleuropein diglucoside isomer c	3.32 ± 0.33 a	1.86 ± 0.14 b
Oleuropein diglucoside isomer d	3.30 ± 0.28 a	2.81 ± 0.22 b
Oleuropein isomer a	5.25 ± 0.51 a	4.35 ± 0.39 b
Oleuropein isomer b	0.52 ± 0.05 a	0.38 ± 0.04 b
Oleuropein isomer c	2.84 ± 0.27 a	1.98 ± 0.18 b
Oleuropein isomer d	1.43 ± 0.13 a	1.14 ± 0.11 b
Quercetin	0.53 ± 0.04 a	0.32 ± 0.03 b
Rutin	1.00 ± 0.10 a	1.10 ± 0.10 a
Secologanoside	3.02 ± 0.21 a	1.02 ± 0.01 b
Taxifolin	1.24 ± 0.11 a	0.57 ± 0.05 b
Verbascoside	3.98 ± 0.30 a	1.87 ± 0.18 b

Different letters (a,b) in the same line indicate significant (*p* < 0.05) differences.

## Data Availability

The original contributions presented in the study are included in the article/supplementary material, further inquiries can be directed to the corresponding author.

## Supplementary Materials

The following supporting information can be downloaded at: https://www.mdpi.com/article/10.3390/foods14030368/s1, Table S1: Standards used for the tentative quantification of phenolic compounds.

## References

[1] Sánchez-Gutiérrez M., Bascón-Villegas I., Rodríguez A., Pérez-Rodríguez F., Fernández-Prior Á., Rosal A., Carrasco E. (2021). Article valorisation of *Olea europaea* L. Olive leaves through the evaluation of their extracts: Antioxidant and antimicrobial activity. Foods.

[2] Benavente-García O., Castillo J., Lorente J., Ortuño A., Del Rio J.A. (2000). Antioxidant activity of phenolics extracted from *Olea europaea* L. leaves. Food Chem..

[3] Lockyer S., Rowland I., Spencer J.P.E., Yaqoob P., Stonehouse W. (2017). Impact of phenolic-rich olive leaf extract on blood pressure, plasma lipids and inflammatory markers: A randomised controlled trial. Eur. J. Nutr..

[4] Ghanbari R., Anwar F., Alkharfy K.M., Gilani A.H., Saari N. (2012). Valuable nutrients and functional bioactives in different parts of olive (*Olea europaea* L.)—A review. Int. J. Mol. Sci..

[5] Sanchez-Rodriguez E., Biel-Glesson S., Fernandez-Navarro J.R., Calleja M.A., Espejo-Calvo J.A., Gil-Extremera B., La Torre R.D., Fito M., Covas M.I., Vilchez P. (2019). Effects of Virgin Olive Oils Differing in Their Bioactive Compound Contents on Biomarkers of Oxidative Stress and Inflammation in Healthy Adults: A Randomized Double-Blind Controlled Trial. Nutrients.

[6] Mylonaki S., Kiassos E., Makris D.P., Kefalas P. (2008). Optimisation of the extraction of olive (*Olea europaea*) leaf phenolics using water/ethanol-based solvent systems and response surface methodology. Anal. Bioanal. Chem..

[7] Dai J., Mumper R.J. (2010). Plant phenolics: Extraction, analysis and their antioxidant and anticancer properties. Molecules.

[8] Barba F.J., Parniakov O., Pereira S.A., Wiktor A., Grimi N., Boussetta N., Saraiva J.A., Raso J., Martin-Belloso O., Witrowa-Rajchert D. (2015). Current applications and new opportunities for the use of pulsed electric fields in food science and industry. Food Res. Int..

[9] Deng Y., Zhou J., Wang B., Xu X., Huang T., Xu Z., Zhao C. (2024). Optimization of Different Extraction Methods for Phenolic Compound Verbascoside from Chinese *Olea europaea* Leaves Using Deep Eutectic Solvents: Impact on Antioxidant and Anticancer Activities. Molecules.

[10] Mir-Cerdà A., Granados M., Saurina J., Sentellas S. (2024). Olive tree leaves as a great source of phenolic compounds: Comprehensive profiling of NaDES extracts. Food Chem..

[11] Cubero-Cardoso J., Hernández-Escaño M., Trujillo-Reyes Á., Fermoso F.G., Fernández-Recamales M.Á., Fernández-Bolaños J., Rodríguez-Gutiérrez G., Urbano J. (2025). Mechanochemical-assisted Natural Deep Eutectic Solvent as a platform for an olive leaves biorefinery: Extraction of bioactive compounds and methane production. Sustain. Chem. Pharm..

[12] Kyriakoudi A., Mourtzinos I., Tyśkiewicz K., Milovanovic S. (2024). An Eco-Friendly Supercritical CO_2_ Recovery of Value-Added Extracts from *Olea europaea* Leaves. Foods.

[13] Nida R., Javad S., Jabeen K., Akhtar I., Kousar N., Shah A.A., Jan M. (2024). Optimization of rapid protocol for extraction of phytochemicals from *Olea europaea* L.—Oleaceae. Pak. J. Bot..

[14] Zayed A., Zahran H.A., Li Z., Khalifa I., Serag A., Fayek N.M., Nicolescu A., Mocan A., Capanoglu E., Farag M.A. (2024). Olive solid wastes: UHPLC-MS/MS-based biochemometric approach for investigating the effect of conventional versus modern extraction methods on in vitro antioxidant, α-glucosidase, and lipase actions. Food Biosci..

[15] Toepfl S., Heinz V., Knorr D. (2007). High intensity pulsed electric fields applied for food preservation. Chem. Eng. Process. Process Intensif..

[16] Razola-Díaz M.d.C., Tylewicz U., Rocculi P., Verardo V. (2023). Application of pulsed electric field processing in the food industry. Non-Thermal Food Processing Operations.

[17] Gachovska T.K., Subbiah J., Thippareddi H., Marx D., Williams F. Inactivation of *E. coli* affected by medium conductivity in pulsed electric field. Proceedings of the 2013 19th IEEE Pulsed Power Conference (PPC).

[18] Corrales M., Toepfl S., Butz P., Knorr D., Tauscher B. (2008). Extraction of anthocyanins from grape by-products assisted by ultrasonics, high hydrostatic pressure or pulsed electric fields: A comparison. Innov. Food Sci. Emerg. Technol..

[19] Ahmad Shiekh K., Odunayo Olatunde O., Zhang B., Huda N., Benjakul S. (2021). Pulsed electric field assisted process for extraction of bioactive compounds from custard apple (*Annona squamosa*) leaves. Food Chem..

[20] Athanasiadis V., Lakka A., Palaiogiannis D., Pappas V.M., Bozinou E., Ntourtoglou G., Makris D.P., Dourtoglou V.G., Lalas S.I. (2021). Pulsed electric field and *Salvia officinalis* L. Leaves: A successful combination for the extraction of high value added compounds. Foods.

[21] Bachtler S., Bart H.J. (2018). Polyphenols from red vine leaves using alternative processing techniques. Processes.

[22] Bozinou E., Karageorgou I., Batra G., Dourtoglou V.G., Lalas S.I. (2019). Pulsed electric field extraction and antioxidant activity determination of moringa oleifera dry leaves: A comparative study with other extraction techniques. Beverages.

[23] Segovia F.J., Luengo E., Corral-Pérez J.J., Raso J., Almajano M.P. (2015). Improvements in the aqueous extraction of polyphenols from borage (*Borago officinalis* L.) leaves by pulsed electric fields: Pulsed electric fields (PEF) applications. Ind. Crops Prod..

[24] Fincan M. (2015). Extractability of phenolics from spearmint treated with pulsed electric field. J. Food Eng..

[25] Razola-Díaz M.d.C., Sevenich R., Rossi Ribeiro L., Guerra-Hernández E.-J., Schlüter O., Verardo V. (2024). Combined effect of pulse electric field and probe ultrasound technologies for obtaining phenolic compounds from orange by-product. LWT.

[26] Razola-Díaz M.d.C., Genovese J., Tylewicz U., Guerra-Hernández E.J., Rocculi P., Verardo V. (2024). Enhanced extraction of procyanidins from avocado processing residues by pulsed electric fields pre-treatment. LWT.

[27] Wang L., Boussetta N., Lebovka N., Vorobiev E. (2020). Cell disintegration of apple peels induced by pulsed electric field and efficiency of bio-compound extraction. Food Bioprod. Process..

[28] Delsart C., Cholet C., Ghidossi R., Grimi N., Gontier E., Gény L., Vorobiev E., Mietton-Peuchot M. (2014). Effects of Pulsed Electric Fields on Cabernet Sauvignon Grape Berries and on the Characteristics of Wines. Food Bioprocess Technol..

[29] Ntourtoglou G., Drosou F., Chatzimitakos T., Athanasiadis V., Bozinou E., Dourtoglou V.G., Elhakem A., Sami R., Ashour A.A., Shafie A. (2022). Combination of Pulsed Electric Field and Ultrasound in the Extraction of Polyphenols and Volatile Compounds from Grape Stems. Appl. Sci..

[30] Boussetta N., Lesaint O., Vorobiev E. (2013). A study of mechanisms involved during the extraction of polyphenols from grape seeds by pulsed electrical discharges. Innov. Food Sci. Emerg. Technol..

[31] Carpentieri S., Ferrari G., Pataro G. (2022). Optimization of Pulsed Electric Fields-Assisted Extraction of Phenolic Compounds From White Grape Pomace Using Response Surface Methodology. Front. Sustain. Food Syst..

[32] Brianceau S., Turk M., Vitrac X., Vorobiev E. (2015). Combined densification and pulsed electric field treatment for selective polyphenols recovery from fermented grape pomace. Innov. Food Sci. Emerg. Technol..

[33] Castagnini J.M., Iaccheri E., Tylewicz U., Dalla Rosa M., Rocculi P. (2020). Pulsed electric fields effect on mechanical and sorption properties of dried apple tissue. Innov. Food Sci. Emerg. Technol..

[34] Pollini L., Cossignani L., Juan C., Mañes J. (2021). Extraction of phenolic compounds from fresh apple pomace by different non-conventional techniques. Molecules.

[35] Lammerskitten A., Mykhailyk V., Wiktor A., Toepfl S., Nowacka M., Bialik M., Czyżewski J., Witrowa-Rajchert D., Parniakov O. (2019). Impact of pulsed electric fields on physical properties of freeze-dried apple tissue. Innov. Food Sci. Emerg. Technol..

[36] Zhang C., Lyu X., Arshad R.N., Aadil R.M., Tong Y., Zhao W., Yang R. (2023). Pulsed electric field as a promising technology for solid foods processing: A review. Food Chem..

[37] Martín-García B., De Montijo-Prieto S., Jiménez-Valera M., Carrasco-Pancorbo A., Ruiz-Bravo A., Verardo V., Gómez-Caravaca A.M. (2022). Comparative Extraction of Phenolic Compounds from Olive Leaves Using a Sonotrode and an Ultrasonic Bath and the Evaluation of Both Antioxidant and Antimicrobial Activity. Antioxidants.

[38] Talhaoui N., Gómez-Caravaca A.M., León L., De la Rosa R., Segura-Carretero A., Fernández-Gutiérrez A. (2014). Determination of phenolic compounds of “Sikitita” olive leaves by HPLC-DAD-TOF-MS. Comparison with its parents “Arbequina” and “Picual” olive leaves. LWT.

[39] Cardoni M., Olmo-García L., Serrano-García I., Carrasco-Pancorbo A., Mercado-Blanco J. (2023). The roots of olive cultivars differing in tolerance to Verticillium dahliae show quantitative differences in phenolic and triterpenic profiles. J. Plant Interact..

[40] López-Salas L., Díaz-Moreno J., Ciulu M., Borrás-Linares I., Quirantes-Piné R., Lozano-Sánchez J. (2024). Monitoring the Phenolic and Terpenic Profile of Olives, Olive Oils and By-Products throughout the Production Process. Foods.

[41] Grigoletto I., García Salas P., Valli E., Bendini A., Ferioli F., Pasini F., Sánchez Villasclaras S., García-Ruiz R., Gallina Toschi T. (2024). HPLC-MS/MS Phenolic Characterization of Olive Pomace Extracts Obtained Using an Innovative Mechanical Approach. Foods.

[42] Brand-Williams W., Cuvelier M.E., Berset C. (1995). Use of a free redical method to evaluate antioxidant activity. LWT Food Sci. Technol..

[43] Parejo I., Codina C., Petrakis C., Kefalas P. (2000). Evaluation of scavenging activity assessed by Co(II)/EDTA-induced luminol chemiluminescence and DPPH· (2,2-diphenyl-1-picrylhydrazyl) free radical assay. J. Pharmacol. Toxicol. Methods.

[44] Alexandre E.M.C., Castro L.M.G., Moreira S.A., Pintado M., Saraiva J.A. (2017). Comparison of Emerging Technologies to Extract High-Added Value Compounds from Fruit Residues: Pressure- and Electro-Based Technologies. Food Eng. Rev..

[45] Montgomery D., St C. (2022). Design and Analysis of Experiments, 9th ed.

[46] Raso J., Frey W., Ferrari G., Pataro G., Knorr D., Teissie J., Miklavčič D. (2016). Recommendations guidelines on the key information to be reported in studies of application of PEF technology in food and biotechnological processes. Innov. Food Sci. Emerg. Technol..

[47] Debs E., Abi-Khattar A.M., Rajha H.N., Abdel-Massih R.M., Assaf J.C., Koubaa M., Maroun R.G., Louka N. (2023). Valorization of Olive Leaves through Polyphenol Recovery Using Innovative Pretreatments and Extraction Techniques: An Updated Review. Separations.

[48] Pappas V.M., Lakka A., Palaiogiannis D., Athanasiadis V., Bozinou E., Ntourtoglou G., Makris D.P., Dourtoglou V.G., Lalas S.I. (2021). Optimization of pulsed electric field as standalone “green” extraction procedure for the recovery of high value-added compounds from fresh olive leaves. Antioxidants.

[49] Tsevdou M., Ntzimani A., Katsouli M., Dimopoulos G., Tsimogiannis D., Taoukis P. (2024). Comparative Study of Microwave, Pulsed Electric Fields, and High Pressure Processing on the Extraction of Antioxidants from Olive Pomace. Molecules.

[50] Moghaddam T.N., Elhamirad A.H., Saeidi Asl M.R., Shahidi Noghabi M. (2020). Pulsed electric field-assisted extraction of phenolic antioxidants from tropical almond red leaves. Chem. Pap..

[51] Chatzimitakos T., Athanasiadis V., Kalompatsios D., Kotsou K., Mantiniotou M., Bozinou E., Lalas S.I. (2024). Optimizing Extract Preparation from Laurel (*Laurus nobilis* L.) Leaves Using a Pulsed Electric Field. ChemEngineering.

[52] Tzima K., Brunton N.P., Lyng J.G., Frontuto D., Rai D.K. (2021). The effect of Pulsed Electric Field as a pre-treatment step in Ultrasound Assisted Extraction of phenolic compounds from fresh rosemary and thyme by-products. Innov. Food Sci. Emerg. Technol..

[53] Talhaoui N., Gómez-Caravaca A.M., Roldán C., León L., De la Rosa R., Fernández-Gutiérrez A., Segura-Carretero A. (2015). Chemometric Analysis for the Evaluation of Phenolic Patterns in Olive Leaves from Six Cultivars at Different Growth Stages. J. Agric. Food Chem..

[54] Coppa C.F.S.C., Gonçalves B.L., In Lee S.H., Nunes V.M.R., Gonçalves C.B., Rodrigues C.E.C., Oliveira C.A.F. (2020). Extraction of oleuropein from olive leaves and applicability in foods. Qual. Assur. Saf. Crops Foods.

[55] Martín-García B., Tylewicz U., Verardo V., Pasini F., Gómez-Caravaca A.M., Caboni M.F., Dalla Rosa M. (2020). Pulsed electric field (PEF) as pre-treatment to improve the phenolic compounds recovery from brewers’ spent grains. Innov. Food Sci. Emerg. Technol..

[56] Hassen I., Casabianca H., Hosni K. (2015). Biological activities of the natural antioxidant oleuropein: Exceeding the expectation—A mini-review. J. Funct. Foods.

[57] Bertelli M., Kiani A.K., Paolacci S., Manara E., Kurti D., Dhuli K., Bushati V., Miertus J., Pangallo D., Baglivo M. (2020). Hydroxytyrosol: A natural compound with promising pharmacological activities. J. Biotechnol..

[58] Gallardo-Fernández M., Gonzalez-Ramirez M., Cerezo A.B., Troncoso A.M., Garcia-Parrilla M.C. (2022). Hydroxytyrosol in Foods: Analysis, Food Sources, EU Dietary Intake, and Potential Uses. Foods.

[59] Alipieva K., Korkina L., Orhan I.E., Georgiev M.I. (2014). Verbascoside—A review of its occurrence, (bio)synthesis and pharmacological significance. Biotechnol. Adv..

