# Supplementary material for: Improving Olive Leaf Phenolic Extraction with Pulsed Electric Field Technology Pre-Treatment
Source: Foods. 2025 Jan 23;14(3):368. doi: 10.3390/foods14030368 (PMC11816887; doi:10.3390/foods14030368)
Supplement: Supplementary file 1 [file foods-14-00368-s001.zip › foods-3387023-supplementary.pdf]

Table S1. Standards used for the tentative quantification of phenolic compounds.

Compound	Quantification standard used
2''-Methoxyoleuropein isomer a	Oleuropein
2''-Methoxyoleuropein isomer b	Oleuropein
2''-Methoxyoleuropein isomer c	Oleuropein
Apigenin glucoside	Apigenin
Apigenin rutinoside	Apigenin
Caffeic acid derivate isomer a	Chlorogenic acid
Caffeic acid derivate isomer b	Chlorogenic acid
Caffeic alcohol derivative	Chlorogenic acid
Chrysoeriol glucoside isomer a	Rutin
Chrysoeriol glucoside isomer b	Rutin
Demethyloleuropein	Oleuropein
Diosmin	Rutin
Hydroxyoleuropein isomer a	Oleuropein
Hydroxyoleuropein isomer b	Oleuropein
Hydroxyoleuropein isomer c	Oleuropein
Hydroxyoleuropein isomer d	Oleuropein
Hydroxytyrosol	Hydroxityrosol
Ligstroside isomer a	Oleuropein
Ligstroside isomer b	Oleuropein
Lucidumoside C isomer a	Oleuropein
Lucidumoside C isomer b	Oleuropein
Lucidumoside C isomer c	Oleuropein
Lucidumoside C isomer d	Oleuropein
Luteolin	Apigenin
Luteolin diglucoside	Apigenin
Luteolin glucoside isomer a	Apigenin
Luteolin glucoside isomer b	Apigenin
Luteolin glucoside isomer c	Apigenin
Luteolin glucoside isomer d	Apigenin
Luteolin rutinoside	Apigenin
Oleoside	Oleuropein
Oleoside methyl ester isomer a	Oleuropein
Oleoside methyl ester isomer b	Oleuropein
Oleuropein aglycon	Oleuropein
Oleuropein diglucoside isomer a	Oleuropein
Oleuropein diglucoside isomer b	Oleuropein
Oleuropein diglucoside isomer c	Oleuropein
Oleuropein diglucoside isomer d	Oleuropein
Oleuropein isomer a	Oleuropein
Oleuropein isomer b	Oleuropein
Oleuropein isomer c	Oleuropein
Oleuropein isomer d	Oleuropein
Quercetin	Rutin
Rutin	Rutin

Secologanoside	Oleuropein
Taxifolin	Rutin
Verbascoside	Hydroxityrosol
